# Validation and Application of a Spanish Version of the ALR-RSI Scale (Ankle Ligament Reconstruction—Return to Sport after Injury) in an Active Non-Athlete Population

**DOI:** 10.3390/jpm13040606

**Published:** 2023-03-30

**Authors:** Sagrario Pérez- de la Cruz

**Affiliations:** Department of Nursing, Physiotherapy and Medicine, University of Almería, 04120 La Cañada de San Urbano, AL, Spain; spd205@ual.es; Tel.: +34-950214574

**Keywords:** ankle, ligament, reconstruction, scale, validation

## Abstract

The most recent scale to quantify psychological readiness before returning to sport is the ALR-RSI (Ankle Ligament Reconstruction—Return to Sport after Injury) scale. The aim of this study was the cross-cultural adaptation to Spanish and application of the ALR-RSI scale in a sample of active people who were not professional athletes, and to carry out an initial psychometric analysis of the functioning of the instrument in this sample. The sample consisted of 257 participants (161 men and 96 women) aged between 18 and 50 years. The adequacy of the model obtained in the exploratory study was confirmed, obtaining a model composed of one factor and 12 indicators in total. The estimated parameters were statistically significant (*p* < 0.05), and the factor loadings presented values higher than 0.5; thus, all indicators revealed a satisfactory saturation in the latent variable (convergent validity). Regarding internal consistency, the Cronbach’s alpha value was 0.886 (excellent internal consistency). This study demonstrated that the ALR-RSI in Spanish is a valid and reproducible scale for evaluating psychological readiness to return to non-professional physical activity after ankle ligament reconstruction in the Spanish population.

## 1. Introduction

Ankle sprains are defined as the stretching, partial rupture, or complete rupture of at least one ligament in the ankle region [1]. Ankle sprains are common injuries that affect the general population, with an incidence of one case per 10,000 people per day [2]. Up to 70% of the general population report having incurred an ankle injury during their lifetime [3], and these lesions are particularly prevalent in athletes. Notably, basketball is the sport most frequently associated with this condition [2].

Most ankle sprains affect the lateral ankle ligaments, following trauma in ankle inversion and plantar flexion, where 70% of cases occur as an isolated injury to the anterior talofibular ligament [4]. The medial collateral ligaments are stronger; therefore, this region is less frequently affected, and when it is, it is usually associated with a more violent trauma in ankle eversion and external rotation of the foot. The mechanism of injury is similar for both the anterior and posterior talofibular ligaments; therefore, both injuries often occur simultaneously [4,5].

In people with a sedentary lifestyle, these injuries may be less disabling. However, in athletes and people whose work demands greater physical exertion, such injuries can have permanent and more disabling consequences [3,4]. Some sports practices, such as basketball, soccer, or volleyball, have a high incidence of ankle injuries, representing approximately 10–15% of sports-related injuries [2,6].

Lateral ankle sprain injuries have the highest reinjury rate of all lower-limb musculoskeletal injuries [7]. Individuals who incur an acute lateral ankle sprain injury have a twofold increased risk of reinjury in the year following their initial injury [8]. Reinjury coincides with the progression of a number of chronic-injury-associated sequelae.

The National Athletic Trainers’ Association (NATA) [9] has approved some documents providing clinicians with comprehensive evidence-based recommendations, and each one highlights the need for assessing patient-reported outcomes in the management of ankle sprains. Patient-reported outcomes are outcomes that are important and meaningful to the patient and should inform the approach to patient care, including return-to-sport decisions. The current best return-to-sport practices recommend a shared decision-making approach that incorporates both biological (structure and function) and psychological (tolerance, fear) assessments when making a return-to-sport decision [10].

An array of social aspects or psychological obstacles—such as fear of reinjury—are factors worth considering when returning to sports practice [11,12,13,14]. Thus, psychological factors will always influence a trauma or physical injury, and most athletes with injuries will experience negative feelings and lack of confidence before returning to practice, due to their decreased physical capacity [15]. 

It is important to know whether the fear of injury persists beyond the initial phase of return to practice in order to maximize participation in the medium and long term. Thus, fear plays an important role in determining an individual’s social and emotional wellbeing [16,17,18]. Nonetheless, few studies have provided a mid–long-term follow-up of athletes who have suffered a major injury requiring a prolonged period of rehabilitation and sports absence [15]. It is important to highlight the importance of the psychological factors involved during the rehabilitation process. Patients faced with the same injury and the same rehabilitation process have shown a lack of adherence, motivation, and self-confidence during the rehabilitation period, and they have been harmed both in their evolution and in their intervention and recovery time. Junge [19] suggested that if these psychological problems were controlled, the persistence of negative symptoms and adherence would be resolved. To further explore this issue, several assessment tools have been developed to evaluate these factors, including, among others, the Tampa Kinesiophobia Scale (TSK) [20]—a validated 11-item questionnaire that assesses fear of movement and injury relapse in people who play sports, with higher scores representing increased kinesiophobia; the Knee Self-Efficacy Scale (K-SES) [21], which is a 22-item questionnaire that measures self-efficacy after ligament injury; the Pain Self-Efficacy Questionnaire (PSEQ) [22], which measures an individual’s perceived ability to perform a series of activities or tasks; the Emotional Response of Athletes to Injury Questionnaire (ERAIQ) [18]; the Sport Rehabilitation Locus of Control (SRLC) scale [23]; and, more recently, the Ankle Ligament Reconstruction—Return to Sport after Injury (ACL-RSI) scale [24].

The aim of this study was to carry out a cross-cultural adaptation to the Spanish language and application of the ALR-RSI scale in a sample of active non-professional athletes from different geographic locations in Spain. The secondary objective was to perform an initial psychometric analysis of this instrument applied to this sample and its validation.

## 2. Materials and Methods

### 2.1. Participants

The sample consisted of 257 participants (161 men and 96 women) who were active people, practicing some type of sports activity, but at neither a competitive nor professional level, and who had suffered an ankle injury at some point. A questionnaire was used that included a series of demographic variables, such as sex, age, level of education, time spent practicing sports, perception of physical fitness, and health status. All of the participants resided in Spain and had a perfect understanding of spoken and written Spanish.

### 2.2. Scale

The Ankle Ligament Reconstruction Scale—Return to Sport after Injury (ALR-RSI) scale is a valid and reproducible scale that helps to identify patients who are ready to resume the same sport that caused the ankle ligament injury after ankle ligament reconstruction. This scale can help predict whether athletes may encounter difficulties in resuming their sporting activities [24,25].

The ALR-RSI scale is based on three components that have been shown to be associated with return to sport: emotions, confidence in performance, and evaluation of risk [20]. Twelve questions are included, with an 11-point Likert scale (highest to lowest) for scoring from 0 to 10. The total score is calculated by adding the values of the 12 responses and then dividing the result by 1.2 to obtain a percentage. High scores correspond to positive psychological responses. The total score is determined by summing the values of the 12 responses and then calculating their relationship to 100 to obtain a percentage.

This scale is validated in different languages, always focused on competitive athletes, although not focused on active people who have suffered this type of injury and have required intervention by a professional for readaptation to physical activity.

#### Procedure

Ethical approval for this study was granted by the Bioethics Committee of the University of Almeria (Ref: UALBIO2020/022). This study was conducted between September and December 2021. 

The instrument was administered by personal and/or virtual interview. The interviewer noted the answers given by the respondent. All participants were informed of the purpose of the study, of their autonomy, and of the absolute confidentiality of their responses and the management of the data obtained. They were also informed that there were no right or wrong answers and were asked to respond with utmost sincerity and honesty.

Following Hambleton’s guidelines [16], a back-translation strategy was adopted. The scale was translated into Spanish and, later, another group translated the scale back into English and compared it with the original. In this process of comparison, the two scales were found to coincide. Subsequently, the battery of items was subjected to an evaluation by three experts on the subject who were external to the study. They considered that the items were pertinent to measure what was intended and confirmed that the wording was correct.

The Spanish version of the ALR- RSI scale is shown below.

La reconstrucción del ligamento del tobillo-regreso al deporte después de una lesión

ALR-RSI (ESCALA DE VALORACIÓN)

1. ¿Está seguro de que puede practicar deporte en un nivel anterior a la lesión?
  Nada seguro (0)…totalmente seguro (10)
2. ¿Cree que es probable que vuelva a lesionarse el tobillo al practicar algún deporte?
  Muy probable (0)…no es probable en absoluto (10)
3. ¿Está nervioso por practicar deporte?
  Extremadamente nervioso (0)…en absoluto nervioso (10)
4. ¿Estás seguro de que su tobillo no cederá al practicar su actividad deportiva?
  Nada seguro (0)…Totalmente confiado (10)
5. ¿Estás seguro de que podría practicar su deporte sin preocuparse por su tobillo?
  Nada seguro (0)…Totalmente confiado (10)
6. ¿Le resulta frustrante tener que estar pendiente de su tobillo con respecto a la práctica deportiva (por si vuelve a ocasionar problemas)?
  Extremadamente frustrante (0)…Nada frustrante (10)
7. ¿Tiene miedo de volver a lesionarse el tobillo practicando su deporte?
  Miedo extremo (0)…Sin miedo en absoluto (10)
8. ¿Confía en que su tobillo resista bajo presión?
  Nada seguro (0)…Totalmente confiado (10)
9. ¿Tiene miedo de lesionarse accidentalmente el tobillo al practicar su deporte?
  Extremadamente asustado (0)…No tengo miedo (10)
10. ¿La idea de tener que someterse a una cirugía y rehabilitación le impide practicar su deporte?
  Todo el tiempo (0)…Ninguna vez (10)
11. ¿Confía en su capacidad para la práctica deportiva?
  Nada seguro (0)…Totalmente confiado (10)
12. ¿Se siente relajado/a al practicar deporte?
  Nada relajado (0)…Completamente relajado (10)

Puntuación total ALR-RSI × 100/120 = __%

Before administering the scale to the final sample, the complete scale was first administered to a sample of 40 participants, confirming that no comprehension problems were observed, after which the final scale was administered to the final study sample. 

In the final administration, the participants were informed about how to complete the questionnaire, insisting that they not leave any item unanswered, as well as assured of the anonymity of their answers and the voluntary nature of their participation. The time required to complete the scale was approximately 10 min. 

### 2.3. Statistical Analysis

Qualitative variables were described by absolute (n) and relative (%) frequencies, and the mean and standard deviation (SD) were used for quantitative variables. 

To determine the suitability of the proposed items, as well as the factorial structure of the questionnaire, an exploratory factor analysis was performed using the principal components method with varimax rotation. After the model generated in the exploratory study, a confirmatory analysis with structural equations was performed using the maximum likelihood extraction method.

Model fit was assessed by means of the following fit indices: goodness-of-fit index (GFI), adjusted goodness-of-fit index (AGFI), comparative fit index (CFI), normed fit index (NFI), Tucker–Lewis index (TLI) and root-mean-square error of approximation (RMSEA). Internal consistency was also examined using Cronbach’s alpha, and convergent validity was tested by means of factor loadings.

## 3. Results

The final study sample consisted of 257 participants, 37.4% of whom were women (n = 96) and 62.6% (n = 161) of whom were men, aged between 18 and 50 years, with a mean age of 27.6 years (SD = 9.9). Up to 86.4% had studied at a university, 39.7% exercised for between one and five hours per week, and 70.1% considered that their physical condition was good. Table 1 shows the sociodemographic variables of the study participants. 

### 3.1. Exploratory Factor Analysis (EFA)

The exploratory factor analysis was developed following the principal components methodology with varimax rotation (Table 2). This analysis revealed the existence of a single factor with an eigenvalue greater than 1, explaining 65.76% of the total variance. 

The result of Bartlett’s test of sphericity determined the non-independence of the variables (Bartlett test = 2387.86 (df = 66), *p* < 0.001). Additionally, the Kaiser–Meyer–Olkin (KMO) test, which seeks to show the adequacy of the sample, yielded a result of 0.952, and the communalities were higher than 0.65. Finally, the set of measures of sampling adequacy (MSA) values were above 0.82. In summary, these values support the factor analysis of the correlation matrix. 

### 3.2. Confirmatory Factor Analysis (CFA)

Based on the model generated in the exploratory study, the confirmatory analysis was calculated with structural equations using the maximum likelihood extraction method.

**Figure 1:**
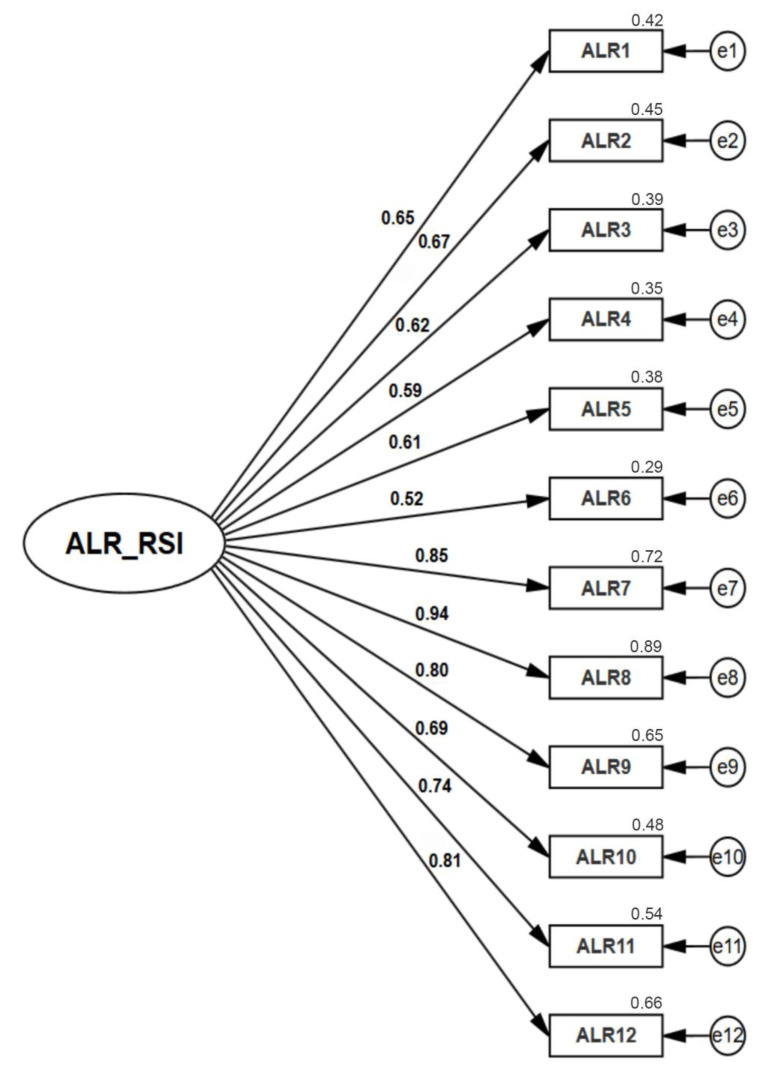
Confirmatory factor analysis (CFA).

The adequacy of the model obtained in the exploratory study was confirmed, since a model composed of one factor and 12 indicators in total was obtained (Figure 1). The estimated parameters were statistically significant (*p* < 0.05), and the factor loadings presented values greater than 0.5; therefore, it can be stated that all the indicators satisfactorily saturated the latent variable (convergent validity). Regarding internal consistency, the Cronbach’s alpha value was 0.886. 

Concerning the model fit (Table 3), the various fit indices were adequate; therefore, the proposed model of the factorial structure of the scale can be considered as largely confirmed.

As shown in Table 4, none of the variables presented skewness values greater than 3 and kurtosis greater than 10, indicating that there were no problems of normality in the observed variables that formed part of the structural equation model. Moreover, the value of the multivariate Mardia index was 32.48, i.e., less than 70, indicating no departure from multivariate normality.

## 4. Discussion

The ALR-RSI scale is the first score that specifically assesses the psychological impact on returning to sports after an ankle ligament reconstruction. The ALR-RSI scale [16], with solid psychometric characteristics, has provided the basis for this validation in Spanish in a specific population. Compared with the 130 volunteers recruited by Thiebat [26], 257 participants from different regions of Spain participated in this study. Thiebat [26], in an attempt to validate the ALR-RSI scale in Italian, concluded that it was a reliable scale for measuring the ability of a person who has suffered an ankle injury to return to any sporting activity involving the damaged joint. It should be noted that among the subjects who participated in Thiebat’s study [26], there were also people from diverse backgrounds—not exclusively from the sports field. This fact confers greater applicability because of having a more varied sample with diverse motivations. This aspect leads to the collection of data related to motivational aspects that can occur in the majority of the population, which is a positive applicability factor for the general implementation of this scale. 

A systematic review published by Ardern et al. [27] analyzed the psychological factors associated with return to sport after injury in people who play sport professionally. They showed that motivation, confidence, and low fear were associated with an increased likelihood of returning to pre-injury levels. On the other hand, fear was the strongest negative emotion that prevented a quick and complete return to sport. It has been shown that patients with positive psychological responses preoperatively and at the time of recovery were associated with a better return to pre-injury levels. 

Recovery was associated with a better return to sport, suggesting that attention to psychological recovery as well as physical recovery may be warranted. Clinical detection of inappropriate psychological responses in athletes may help those at risk of not recovering their level of fitness to recover their sporting level, which presumably could be extrapolated to non-professional sportspeople.

To provide consistency and validity to this scale, which was initially intended for the professional athlete population, in this study we recruited a varied sample that practiced moderate physical activity. A Spanish validation study of the ARL-RSI scale for athletes was previously carried out by Sala-Barat et al. [28], with a sample of 114 professional soccer players. The results of this previous study indicated that the ARL-RSI scale is a valid and reliable instrument to assess the psychological factors relevant to the return to sports practice of soccer players after a lateral ankle ligament injury. Similar results have been obtained in validations of the scale in Italian, Malay, Swedish, Turkish, Chinese, and French, with specific populations and limited numbers of participants [5,13,25,28,29].

The aim of this study was to extend the range and applicability of this scale to other groups who, although not professionally involved in sports, do practice regularly and, therefore, also have high rates of injury in this anatomical structure, as indicated by studies such as those of Doherty et al. [30] and Herzog et al. [31]. The terminology used in the statements has also been considered, so that it is not so specific to the sports field but can be applied and implemented by a broad spectrum of the population. Indeed, the terms that referred to the field of soccer were adapted and made more general so that they could be extrapolated to the general working population.

Among the results obtained in the statistical analysis of the validation study and adaptation to the non-sports environment, the internal consistency coefficient shown in this study was *α* =0.95, whereas the value obtained in the study of Sala-Barat [28] was *α* = 0.9; both of these values are very close to those obtained in the English scale (*α* = 0.96) [32] and the validations in Swedish (*α* = 0.95) [8], Turkish (*α* = 0.88) [25], Chinese (*α* = 0.96) [5], and French (*α* = 0.96) [29]. Likewise, the test–retest correlation coefficient was excellent (0.9) and comparable to the values obtained in previous studies [5,13,24,25,28,29].

The Spanish version of the ACL-RSI scale also showed excellent discriminatory ability, with notable differences between the scores obtained by patients who expressed their intention to return to soccer and those who did not wish to return to play. In the original version of the scale, Webster et al. [24] identified a factorial structure with a single factor explaining 67.8% of the variance. This one-dimensional structure, with a similar percentage of variance explained, has also been observed in the Swedish [5] and Chinese versions [24]. However, the results of the exploratory factor analysis obtained in the sample of this study indicate a factorial configuration composed of two significant and clearly defined components, which we named confidence in performance and fear and insecurity.

Future studies, with varied and larger samples, may allow us to confirm the consistency of the components that comprise this scale. It seems logical that athletes with a good perception of their physical fitness would be prepared to return to practice after an injury [33]. This fact may be related to the real capacity to return to sports practice; from the medical point of view, however, it would be a determining factor to be considered by the medical team when assessing and managing return from injury. A greater confidence in performance is associated with a greater perception of an athlete’s readiness to return to play. This same situation occurred in this study, where the greater a person’s confidence in recovery, the faster their perceived return to activity. Moreover, a negative perception conditions the return to physical activity. Presenting a high value in the item related to fear and insecurity regarding sports practice indicates that individuals will not be ready to return to sport, even if they have recovered their pre-injury physical condition. Extrapolating these results to the clinical practice of health professionals, these aspects should be considered and incorporated in the early stages of the rehabilitation process after an ankle injury, so as not to alter the natural, rapid, and safe evolution of recovery and return to normality. We postulate that different components of psychological readiness will fluctuate over the course of rehabilitation, consistent with injury symptom resolution; specifically, cognitive appraisals of confidence, expectations, and motivations will increase consistent with symptom resolution, while risk appraisals and negative affectivity will decrease [33].

One of the advantages of this scale design is that it is easy for users to implement. The use of a questionnaire with numeric answers makes it possible to simplify the responses, and to quantify the patients’ evolution. Such a questionnaire can be easily used by doctors and surgeons in their daily practice. Indeed, giving a patient permission to return to sport is a difficult decision to make, and there is no consensus on this subject [34]. The purpose of the score is to enable physicians to recognize patients who have psychological factors that prevent them from resuming their activities. Therefore, the practitioners will be able to offer them specific advice to overcome their apprehension. 

Considering previous works carried out with athlete populations [5,13,25,26,27,28,29,32], one of the limitations of previous studies was that most of the participants were male and of a specific age group (young adults), whereas the sample in the present study , in addition to including a larger number of subjects, was more diverse in age and included female participants. This means that the results obtained may have added value in terms of their validity and applicability. Furthermore, it would have been interesting to have been able to extend the study over time, by carrying out assessments beyond six months post-injury. The recommended period for the full recovery of injuries of this type may undergo exceptions in subjects who present complications and when there is a delay in the return to active life and sports practice. 

Now that the scale has been validated, it would be interesting to extrapolate this scale and demonstrate that the ALR-RSI scale can be used by clinicians working in the recovery of lateral ankle ligament injuries in the active population.

## 5. Conclusions

Regardless of the type of injury, there is a clear correlation between the psychological state of a person and the return to sport for injuries that cause a longer absence from sporting activity. Although psychological interventions are not yet part of rehabilitation treatment, they should be considered during the rehabilitation process. The Spanish version of the ALR-RSI scale is a valid and reproducible scale for evaluating psychological factors that are relevant to the return to non-professional sports practice after ankle ligament reconstruction in the Spanish population.

## Figures and Tables

**Table 1:** Descriptive characteristics of the study sample.

	n	%
Academic training		
Vocational training	12	4.7
Secondary studies	23	8.9
University studies	222	86.4
Weekly physical activity (h)		
1–5	102	39.7
6–10	96	37.4
11–15	41	16
16–20	18	7
Fitness		
Good	180	70.1
Average	71	27.6
Poor	6	2.3

**Table 2:** Exploratory factor analysis.

Item	Communality	Factor
ALR1	0.731	0.703
ALR2	0.721	0.756
ALR3	0.722	0.698
ALR4	0.8	0.852
ALR5	0.715	0.771
ALR6	0.659	0.628
ALR7	0.723	0.732
ALR8	0.847	0.817
ALR9	0.671	0.664
ALR10	0.668	0.805
ALR11	0.746	0.864
ALR12	0.863	0.926

**Table 3:** AFC goodness-of-fit indices.

χ^2^ (g.l.)	*p*	χ^2^/g.l.	GFI	AGFI	CFI	NFI	TLI	RMSEA (I.C. 90%)
154.19 (54)	<0.001	2.86	0.97	0.95	0.96	0.94	0.97	0.036 (0.032–0.044)

g.l.: degrees of freedom. Goodness-of-fit index (GFI), adjusted goodness-of-fit index (AGFI), comparative fit index (CFI), normed fit index (NFI), Tucker–Lewis index (TLI), and root-mean-square error of approximation (RMSEA).

**Table 4:** Skewness and kurtosis.

Variable	Skewness	Kurtosis
ALR1	−1.999	3.548
ALR2	−0.065	−1.093
ALR3	−1.402	1.166
ALR4	−0.742	−0.09
ALR5	−1.165	0.918
ALR6	−0.773	−0.713
ALR7	−0.515	−0.833
ALR8	−0.646	−0.243
ALR9	−0.285	−1.286
ALR10	−1.464	0.9
ALR11	−1.527	2.068
ALR12	−1.001	−0.256

## Data Availability

Data is unavailable due to privacy and ethical restriction.
